# Clinicopathological Characteristics and Postoperative Outcomes Following Parotidectomy: A Ten-Year Retrospective Study from a Tertiary Center

**DOI:** 10.3390/diseases14040143

**Published:** 2026-04-11

**Authors:** Mohammad Aljarba, Mishari Alanezi, Majed A. Alali, Azzam Alotaibi, Faisal Alkhunein, Khalid Alqahtani

**Affiliations:** 1 College of Medicine, King Saud University, Riyadh 11411, Saudi Arabia; misharialanezi5@gmail.com (M.A.); majedalali973@gmail.com (M.A.A.); azzamano12@gmail.com (A.A.); famk20@gmail.com (F.A.); 2 Department of Otolaryngology, Head and Neck Surgery, College of Medicine, King Saud University, P.O. Box 245, Riyadh 11411, Saudi Arabia

**Keywords:** parotid gland, parotidectomy, pleomorphic adenoma, facial nerve palsy, Saudi Arabia

## Abstract

**Background/Objective:** The parotid gland is the largest salivary gland, and tumors arising from it exhibit wide histopathological diversity. Management approaches vary according to tumor characteristics and carry a risk of postoperative complications, particularly facial nerve injury. However, local data remain limited. This study aimed to describe the clinicopathological characteristics, surgical approaches, and postoperative outcomes of patients undergoing parotidectomy. **Method:** A retrospective cohort study was conducted at a high-volume tertiary center in Saudi Arabia. All consecutive patients who underwent parotidectomy between June 2015 and January 2025 were included. Demographic data, histopathological diagnoses, surgical procedures and postoperative complications were extracted from electronic medical records. Statistical analyses were performed using SPSS version 26, with A *p*-value of <0.05 considered statistically significant. **Results:** A total of 154 patients were included, with a mean age of 45.2 ± 12.6 years; 61% were male. Benign lesions constituted 87% of cases, with pleomorphic adenoma being the most common histopathological diagnosis. Malignancies accounted for 13% of cases, most frequently mucoepidermoid carcinoma. The most common postoperative complications were facial nerve palsy, followed by sensory numbness. **Conclusions:** The majority of parotid gland tumors in this cohort were benign, with pleomorphic adenoma as the most common histological subtype. Facial nerve palsy and sensory disturbances were the most common postoperative complications. These findings provide valuable local data on parotid gland lesions in Saudi Arabia and support current surgical management practices.

## 1. Introduction

The parotid gland is the largest salivary gland in the body and plays a major role in saliva production. It lies in the periauricular area and is anatomically related to important structures, such as the facial nerve. Salivary gland neoplasms constitute 3% to 4% of all head and neck tumors, with the parotid gland being the most affected gland [1,2,3]. Most of these lesions are benign, with malignancy reported only in 10% to 15% of all cases [1,2]. Pleomorphic adenoma has been reported as the most common histological subtype in multiple previous studies. However, some recent studies have reported an equal or higher frequency of Warthin’s tumor compared to pleomorphic adenoma [3,4,5,6]. Among malignant parotid gland tumors, mucoepidermoid carcinoma was consistently reported as the most common histopathological subtype [7,8]. However, one study reported metastatic squamous cell carcinoma to be the most common malignant type [9].

Management of parotid gland tumors involves multiple surgical approaches, including enucleation, extracapsular dissection, partial or superficial parotidectomy (SP), total parotidectomy (TP), and radical parotidectomy (RP) [10]. The choice of surgical technique depends on the extent and nature of the lesion [11]. Due to the complex anatomical relationship between the facial nerve and the parotid gland, facial nerve paralysis is a major concern following these surgeries, with the risk of injury increasing with more extensive resections. Previous studies have demonstrated that the risk of facial nerve injury ranges from 2.7% to 6% [12,13]. Other minor complications include sialocele, salivary fistula, numbness, wound infection, bleeding, hematoma, seroma, skin flap necrosis, and scar-related complications. A recent systematic review reported the incidence rate of minor complications as follows: hematoma 2.9%, wound infection 2.3%, sialocele 4.5%, salivary fistula 3.1%, flap necrosis 1.7%, scar issues 3.6%, and numbness 33.9% [14]. Although the epidemiology and outcomes of parotid gland tumors have been well described internationally, there is a lack of studies on this area in Saudi Arabia. Therefore, the aim of this study is to provide a comprehensive analysis of tumor types, surgical approaches, incidence and pattern of postoperative complications in a Saudi population.

## 2. Methods

### 2.1. Study Design, Setting, and Population

This retrospective cohort study was conducted at a high-volume tertiary-care academic center in Saudi Arabia, which serves as a referral center for head and neck surgery and oncologic cases from across the region. We included all consecutive patients who underwent parotidectomy between June 2015 and January 2025.

Patients of all age groups and both genders were eligible for inclusion. Cases were identified through the institutional electronic medical record (EMR) system. Patients were excluded if they had incomplete medical records, missing histopathological diagnoses, or insufficient postoperative follow-up data for complication assessment.

The study was approved by the Institutional Review Board (IRB) of the College of Medicine, King Saud University (Project No. E-24-9314). Due to the retrospective nature of the study and the use of anonymized data, the requirement for informed consent was waived.

### 2.2. Data Collection and Variables

Data were extracted from EMRs and recorded in a standardized computerized database. Collected variables included demographic information, tumor-related characteristics, operative details, and postoperative complications.

Histopathological diagnoses were obtained from final pathology reports and classified into benign and malignant categories. Benign tumors included pleomorphic adenoma, Warthin’s tumor, benign cysts, and other rare benign entities. Malignant tumors were further categorized by histological subtype, including mucoepidermoid carcinoma, acinic cell carcinoma, squamous cell carcinoma, metastatic squamous cell carcinoma, and other less common malignancies.

Surgical procedures were categorized based on operative reports into partial parotidectomy, superficial parotidectomy, total parotidectomy, and radical parotidectomy. Length of hospital stay was calculated as the number of days from admission to discharge.

Postoperative complications were documented based on both inpatient and outpatient follow-up records. These included facial nerve palsy (classified as transient or permanent), numbness, seroma, hematoma, wound infection, hemorrhage, wound edema, scarring-related complications, Frey syndrome, and other miscellaneous complications.

### 2.3. Operative Detail

In both benign and malignant cases, a similar initial surgical approach was followed, including a modified Blair incision, elevation of the skin flap, identification and preservation of the greater auricular nerve when feasible, and localization of the facial nerve using anatomical landmarks between the tympanomastoid suture and the mastoid tip at the level of the digastric muscle.

For benign tumors, surgery typically involved superficial parotidectomy with an approximate 1 cm margin where appropriate. In contrast, malignant tumors generally required total parotidectomy, with neck dissection performed when indicated based on oncologic considerations. These details have now been clarified in the revised manuscript.

### 2.4. Statistical Analysis

Data were analyzed using SPSS software version 26. Continuous variables were reported as mean and standard deviation or as a range, while categorical variables were presented as numbers and percentages.

Normality of continuous data was assessed prior to analysis. Comparisons between benign and malignant groups were performed using independent sample tests for continuous variables and chi-square or Fisher’s exact tests for categorical variables, depending on data distribution and cell counts. Similar comparative analyses were conducted between pleomorphic adenoma and Warthin’s tumor subgroups. All tests were two-sided, and a *p*-value < 0.05 was considered statistically significant.

## 3. Results

### 3.1. Patient Demographics and Clinical Characteristics

The present study comprised a total of 154 patients operated for parotidectomy during the period from 2015 to 2025. The mean age of the entire study population was 45.21 years with a standard deviation of 12.55. The age pattern showed that the majority of patients (55.2%) were in the age group of 40–59 years, followed by 33% in the age range of 20–39 years. Sex patterns showed predominance of male patients (61%) compared to females (39%), with significant statistical significance (*p* = 0.006) (Table 1).

### 3.2. Histopathological Diagnosis

As for the diagnostic results, most of the patients had benign lesions (87%), mostly consisting of pleomorphic adenoma, which was the most common benign diagnosis in the present series (68 cases). Other benign diagnoses included Warthin’s tumor (30 cases) and benign cysts (nine cases), in association with a range of rare benign lesions. On the other hand, malignancies were diagnosed in 13% of patients, with mucoepidermoid carcinoma (three cases) and acinic cell carcinoma (two cases) being most common. The balance of malignant cases was represented by much less common types, such as squamous cell carcinoma and metastatic squamous cell carcinoma (Table 2).

### 3.3. Surgical Procedures and Treatment Patterns

**Figure 1:**
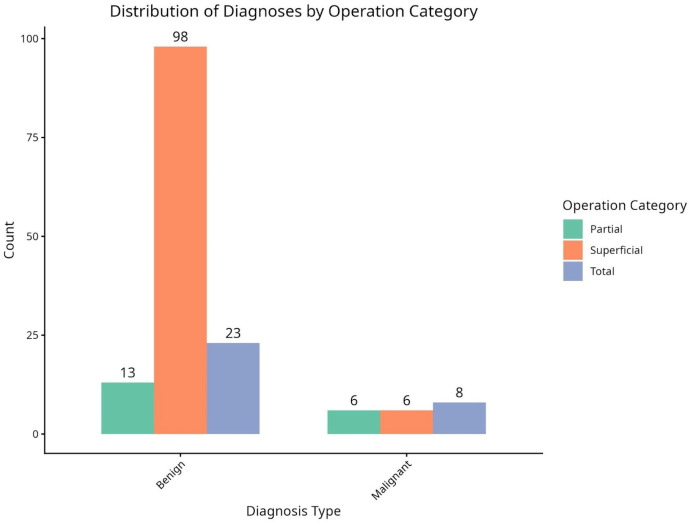
Distribution of diagnosis by operation category.

Regarding surgical procedures, Figure 1 depicts the allocation of operation types for both benign and malignant cases. In the context of benign cases, a predominant majority (98 out of 134) underwent superficial parotidectomy, while partial parotidectomy (13 cases) and total parotidectomy (23 cases) were performed less frequently. Conversely, for malignant cases, there was a more balanced distribution among the various surgical categories, as indicated by the similar proportions of partial parotidectomy (six cases), superficial parotidectomy (six cases), and total parotidectomy (eight cases).

### 3.4. Comparative Analysis Between Benign and Malignant Cases

Comparative study of malignant and benign cases revealed significant differences in hospitalization duration. Average hospitalization time for malignant cases was significantly increased to 2.5 days, compared to 1.34 days for benign cases (*p* = 0.02). No significant differences were recorded among the groups in terms of tumor site (*p* = 0.33), with 53.9% of patients having tumors on their right side and 46.1% on their left side (Table 3).

### 3.5. Clinicopathologic Correlation of Pleomorphic Adenoma and Warthin’s Tumor

Following the analysis of pleomorphic adenoma and Warthin’s tumor classifications, there was a noticeable imbalance in gender allocation, with a higher percentage of males in the Warthin’s tumor classification (*p* < 0.001). Furthermore, the age of pleomorphic adenoma patients was much lower compared with patients with Warthin’s tumor (mean age: pleomorphic adenoma: 39.9 years; Warthin’s tumor: 57.7 years, *p* < 0.001). Although these variations in age were present, time of hospitalization for the two classifications was not significant statistically (*p* = 0.6). The recurrence of pleomorphic adenoma was estimated at 0.059, while that of Warthin’s tumor was recorded as 0.033 (*p* = 0.97) (Table 4).

### 3.6. Postoperative Complications

**Figure 2:**
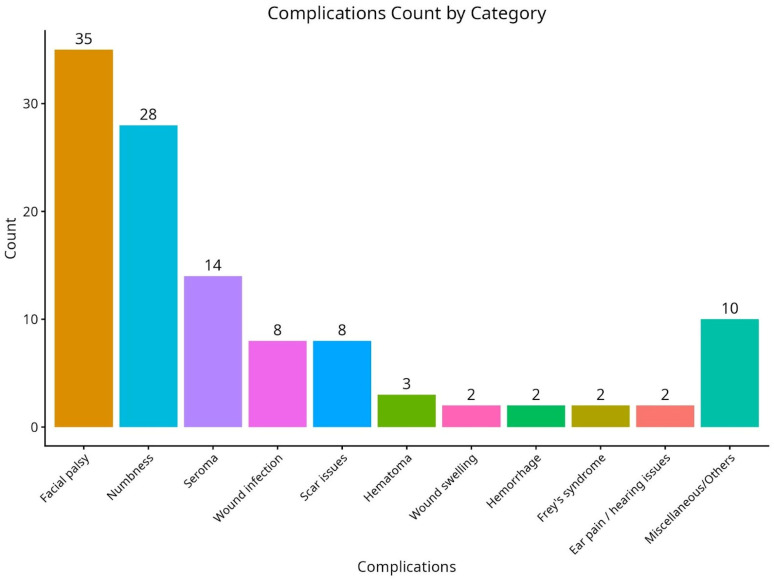
Complication count by category.

All patients were observed for complications, with the most frequent events being facial palsy (35 cases) and numbness (28 cases). Other complications included seroma (14 cases), wound infection (eight cases), and problems with scarring (eight cases). The incidence of more significant complications, including hemorrhage and wound edema, was much lower, with only three and two cases, respectively. Several other complications, grouped as “miscellaneous,” were recorded in 10 cases (Figure 2).

### 3.7. Comparison of Complications Between Benign and Malignant Cases

The comparison of complications between benign and malignant cases showed a higher number of facial palsy cases in the benign group (30 patients) compared with the malignant group (six patients); however, this difference was not statistically significant (*p* = 0.4). Numbness was also observed to happen more in benign cases (16%) in comparison to malignant cases (7.1%). No significant variations were, however, observed in the other complications, including seroma, wound infection, and wound swelling, in the two groups. Conversely, hemorrhage was confirmed to happen more in malignant cases (3.6% compared with 0.6% in benign cases), while some of the other complications were also more common in the malignant series (7.1% compared with 4.8% in benign cases) (Table 5).

## 4. Discussion

The parotid gland shows diverse morphological features and highly varied histological composition, which contribute to extensively varied tumor lesions. This retrospective study aimed to outline a comprehensive analysis of demographic patterns, tumor types, postoperative complications and surgical approaches among patients undergoing parotidectomy.

The findings of this study demonstrate that the vast majority of parotid gland tumors treated at our center were benign, most commonly pleomorphic adenoma, followed by Warthin tumor, while malignant tumors accounted for a smaller proportion of cases. Superficial parotidectomy was the most performed procedure and was the operation most often associated with postoperative facial nerve palsy. These findings closely align with those reported in international studies; moreover, this study helps to address the scarcity of local data, providing valuable insight and contributing to improving the overall patient outcomes.

We observed a predominance of male patients, with males comprising 62% of all cases. This finding is consistent with previous international and local studies, including those by Wong et al. [15] and Salamah et al. [16], both of which reported a clear male predominance among patients with parotid gland tumors. Additionally, our cohort study showed a higher frequency of right-sided tumors, which is consistent with previous reports in the literature [16,17].

The mean age of our study population was 45.21 years, with the majority of patients falling within the 40–59 years age group. Conversely, several international studies have reported substantially higher mean ages [17,18,19]. This discrepancy may be explained by the generally younger population structure in Saudi Arabia, which naturally lowers the average age of presentation, as well as the higher proportion of benign tumors, particularly pleomorphic adenoma, which generally tend to present at a younger age.

This observation aligns with local data from Alqaryan et al. [20], who noted that 42.7% of cases occurred within the 31–50-year age range, underscoring the tendency toward earlier presentation in the local population.

Benign lesions constituted 87% of all diagnoses in our series. Nearly half of these benign lesions were pleomorphic adenomas, which is almost comparable to the 51% reported by Lin et al. [21]. Warthin’s tumor, also known as papillary cystadenoma lymphomatosum, was the second most common benign tumor, which is consistent with previously reported prevalence ranges of 5–20% [22,23,24]. In contrast, Lim et al. was the first to report Warthin’s tumor (40% prevalence) as the most common benign parotid tumor, surpassing pleomorphic adenoma (36% prevalence) in Singapore [25]. The predominance of pleomorphic adenoma observed in our study may be attributed to demographic differences, as pleomorphic adenoma typically presents in younger adults, whereas Warthin’s tumor is more frequently observed among those who are older. Additionally, Warthin’s tumor has a strong association with smoking, and variations in cultural and lifestyle habits, particularly smoking habits, between the two populations may also contribute to this difference [26].

Malignant lesions accounted for 13% of all tumors in our cohort, an incidence comparable to that reported in previously published studies [15,16,17,27]. Mucoepidermoid carcinoma was the most common malignant tumor identified in this study, a finding that is consistent with previous reports [16,21].

When comparing pleomorphic adenoma with Warthin’s tumor, a significant disparity in age and gender distribution was observed. Warthin’s tumor showed a markedly higher rate of male patients and presented at an older mean age, whereas pleomorphic adenoma demonstrated a substantially lower mean age of presentation (39.9 years) compared with Warthin’s tumor (57.7 years). These findings align with those reported by Lin et al. [21], who also observed a statistically significant male predominance and older age at diagnosis with Warthin’s tumor (59 years) compared with pleomorphic adenoma (40 years). This pattern likely reflects the combined effect of age-related changes and cumulative environmental exposures, particularly smoking, which have been strongly associated with the development of Warthin’s tumor in previous studies [26].

The management of parotid gland tumors encompasses a spectrum of approaches, with approaches ranging from systemic treatment for inflammatory conditions to radical surgical intervention for malignant tumors [28]. The extent of surgery depends on several factors, including the patient’s clinical presentation, size of the tumor, tumor location, and histopathological characteristics [18]. Therefore, establishing an accurate diagnosis is crucial to guide appropriate surgical intervention.

From a surgical standpoint, our review showed that in the vast majority of benign cases, patients underwent superficial parotidectomy. This technique was described by O’Brien as “the operation of choice for benign parotid tumors,” as it was found recently that superficial parotidectomy is associated with low morbidity [29]. Conversely, a recent study by Mantsopoulos et al. [30], which included 1624 patients, reported a higher utilization of extracapsular dissection. This trend was attributed to advances in ultrasonographic imaging and increased levels of surgical expertise. Therefore, surgical decision-making should be individualized and based on a personalized, patient-centered approach, taking into account each patient’s clinical status and tumor characteristics, rather than relying on standardized treatment protocols.

Facial nerve palsy emerged as the most common postoperative complication in our study, accounting for 22.1% of cases, including transient palsy in 17% and permanent palsy in 5.1%. This incidence is comparable to the 18% reported in the United States by Upton et al. [31] and falls within the wide ranges described in previous studies which reported facial palsy incidence ranging from 5.2% to 66% [12,32].

Ear numbness was the second most common postoperative complication, accounting for 18.1% of cases, which is similar to previous studies that showed 17.3% in [20] and 20% in [33]. This symptom is likely related to intraoperative manipulation or sacrifice of the auriculotemporal nerve [34]. Thus, patients should be informed of potential sensory disturbances and appropriately counseled regarding these complications. Frey syndrome was observed in 1.2% of patients, a proportion consistent with previously published reports [20,21,30]. For example, Licius et al. reported a 4% incidence in a series of 610 patients [35].

Our study has both strengths and limitations. A key strength is that it includes consecutive parotidectomy cases from a high-volume tertiary referral center identified through the EMR, providing a solid institutional benchmark; future studies should validate these findings through multicenter/national cohorts to improve generalizability. Another strength is the standardized EMR-based data extraction with complication capture from both inpatient and outpatient follow-up; future work should standardize outcome grading/timepoints and add patient-reported measures to improve comparability and capture subjective symptoms. However, the lack of standardized and consistent capture of certain subclassification details across all patients may limit detailed clinical interpretation. Future studies should emphasize systematic subclassification to improve the accuracy and clinical relevance of findings. Moreover, the single-center retrospective design may limit external validity; future research should be prospective and multicenter. Excluding patients with incomplete records or insufficient follow-up may introduce selection bias; future studies should set predefined minimum follow-up and a missing-data plan. The absence of standardized grading and longitudinal follow-up of facial nerve dysfunction represents a limitation of this study. Facial nerve outcomes were not consistently graded using validated tools such as the House–Brackmann scale, and long-term follow-up data were not uniformly available due to the retrospective design. In addition, management approaches for facial nerve dysfunction were not systematically documented, limiting the ability to analyze treatment approaches and their outcomes. Future prospective studies using standardized grading systems and structured follow-up are needed to better evaluate facial nerve management. The small malignant subgroup and inconsistent cohort dates/statistical methods reporting further limit inference; future studies should pool malignant cases across centers and ensure consistent dates with a clearly specified statistical analysis plan.

## 5. Conclusions

This study demonstrates that pleomorphic adenoma was the most common histological subtype. Superficial parotidectomy was the predominant surgical approach and was associated with acceptable morbidity. Facial nerve palsy and sensory disturbances were the most frequent postoperative complications, largely transient, and comparable to rates reported internationally. These findings provide valuable local data on parotid gland tumors in Saudi Arabia and support current surgical management practices, while highlighting the need for further multicenter studies to evaluate long-term outcomes.

## Figures and Tables

**Table 1:** Demographic and clinical characteristics of patients who underwent parotidectomy (2015–2024).

Demographics		Value	Percentage	*p*-Value
**Total Sample Size**	n	154	-	-
**Age**	Whole population (mean SD)	45.21 (12.55)	-	-
	Male age (mean SD)	41.8 (12.1)	-	0.007
	Female age (mean SD)	47.4 (12.4)	-	
**Gender**	Male	94	61%	0.006
	Female	60	39%	
**Age Categories**	0–19	1	0.65%	<0.001
	20–39	51	33%	
	40–59	85	55.2%	
	60–79	16	10.4%	
	80 and older	1	0.65%	
**Diagnosis**	Benign	134	87%	<0.001
	Malignant	20	13%	
**Tumor Site**	Left	71	46.1%	0.33
	Right	83	53.9%	

**Table 2:** Histopathological characteristics of parotid disease.

Benign	N = 134
Pleomorphic adenoma	68
Warthin’s tumor	30
Benign cyst	9
Inflammatory parotid diseases	7
Lipoma	6
Myoepithelioma	4
Reactive lymphoid lesions	3
Basal cell adenoma	2
Cystadenoma	1
Epidermal cyst	1
Lymph epithelial cysts	1
Vascular hemangioma	1
Tenosynovial giant cell tumor	1
**Malignant**	**N = 20**
Mucoepidermoid carcinoma	3
Acinic cell carcinoma	2
Carcinoma ex-pleomorphic adenoma	2
Mammary analogue secretory carcinoma	2
Squamous cell carcinoma	2
Basal cell carcinoma	1
Epithelial–myoepithelial carcinoma	1
High-grade non-Hodgkin lymphoma	1
Hodgkin’s lymphoma	1
MALT lymphoma	1
Metastatic squamous cell carcinoma	1
Parotid acinic cell carcinoma	1
Parotid neuroendocrine carcinoma	1
Poorly differentiated lymphoepithelial carcinoma	1
**Total**	154

**Table 3:** Comparison between benign and malignant cases.

Characteristic	Benign	Malignant	*p*-Value
**No. of patients**	134	20	-
**Male/Female**	82/52	12/8	-
**Age**	44.9 (19–86)	47.6 (22–69)	0.44
**Tumor Site**			1
**Left**	62	9	
**Right**	72	11	
**Average Length of Hospitalization**	1.34	2.5	0.02
**Recurrence Rate**	0.04	0.15	0.2
**Metastasis Rate**	0	0.2	<0.001

**Table 4:** Clinicopathological comparison of pleomorphic adenoma and Warthin’s tumor.

Variables	Pleomorphic Adenoma	Warthin’s Tumor	*p*-Value
**Case No.**	68	30	
**Male/Female**	34/34	30/0	<0.001
**Mean age**	39.9	57.7	<0.001
**Tumor Site**			0.8
**Left**	35	14	
**Right**	33	16	
**Average Length of Hospitalization**	1.46	1.33	0.6
**Recurrence Rate**	0.059	0.033	0.97

**Table 5:** Comparison between benign and malignant cases in terms of complications.

Complications	Benign N = 168	Malignant N = 28	*p*-Value
			0.4
Decrease hearing	0 (0%)	1 (3.6%)	
Ear pain	2 (1.2%)	1 (3.6%)	
Facial palsy	30 (18%)	6 (21%)	
Frey’s syndrome	2 (1.2%)	0 (0%)	
Hematoma	3 (1.8%)	0 (0%)	
Hemorrhage	1 (0.6%)	1 (3.6%)	
Miscellaneous/Others	8 (4.8%)	2 (7.1%)	
None	66 (39%)	11 (39%)	
Numbness	27 (16%)	2 (7.1%)	
Scar issues	6 (3.6%)	2 (7.1%)	
Seroma	14 (8.3%)	1 (3.6%)	
Wound infection	7 (4.2%)	1 (3.6%)	
Wound swelling	2 (1.2%)	0 (0%)	

## Data Availability

The data that support the findings of this study are available from the corresponding author upon reasonable request.
